# The Potential of the FSP1cre-*Pparb/d^−/−^* Mouse Model for Studying Juvenile NAFLD

**DOI:** 10.3390/ijms20205115

**Published:** 2019-10-15

**Authors:** Jiapeng Chen, Yan Zhuang, Ming Keat Sng, Nguan Soon Tan, Walter Wahli

**Affiliations:** 1 Lee Kong Chian School of Medicine, Nanyang Technological University Singapore, 11 Mandalay Road, Singapore 308232, Singapore; chenjiapengkelvin@gmail.com (J.C.); zhuangyan1986@hotmail.com (Y.Z.); mingkeat.sng@gmail.com (M.K.S.); NSTAN@ntu.edu.sg (N.S.T.); 2 School of Biological Sciences, Nanyang Technological University Singapore, 60 Nanyang Drive, Singapore 637551, Singapore; 3 INRA UMR1331, ToxAlim, 180 Chemin de Tournefeuille, 31300 Toulouse, France; 4 Center for Integrative Genomics, University of Lausanne, Le Génopode, CH-1015 Lausanne, Switzerland

**Keywords:** *Pparb/d*, FSP1, lipid metabolism, steatosis, fatty acid β-oxidation, fatty acid synthesis and triglyceride synthesis

## Abstract

Non-alcoholic fatty liver disease (NAFLD) can progress from steatosis to non-alcoholic steatohepatitis (NASH) characterized by liver inflammation, possibly leading to cirrhosis and hepatocellular carcinoma (HCC). Mice with impaired macrophage activation, when fed a high-fat diet, develop severe NASH. Evidence is mounting that Kupffer cells are implicated. However, it is unknown whether the resident CD68^+^ or bone marrow-derived CD11b^+^ Kupffer cells are involved. Characterization of the FSP1cre-*Pparb/d^−/−^* mouse liver revealed that FSP1 is expressed in CD11b^+^ Kupffer cells. Although these cells only constitute a minute fraction of the liver cell population, *Pparb/d* deletion in these cells led to remarkable hepatic phenotypic changes. We report that a higher lipid content was present in postnatal day 2 (P2) FSP1cre-*Pparb/d^−/−^* livers, which diminished after weaning. Quantification of total lipids and triglycerides revealed that P2 and week 4 of age FSP1cre-*Pparb/d^−/−^* livers have higher levels of both. qPCR analysis also showed upregulation of genes involved in fatty acid β-oxidation, and fatty acid and triglyceride synthesis pathways. This result is further supported by western blot analysis of proteins in these pathways. Hence, we propose that FSP1cre-*Pparb/d^−/−^* mice, which accumulate lipids in their liver in early life, may represent a useful animal model to study juvenile NAFLD.

## 1. Introduction

Non-alcoholic fatty liver disease (NAFLD) is an emerging epidemic disease posing a major health issue worldwide. It is the most common cause of liver disease in humans. NAFLD has high prevalence ranging from 11% to 46% in developed countries [1,2,3]. NAFLD is an inclusive term that is used to cover a range of liver pathologies. It begins with steatosis, where lipid accumulation in the hepatocytes is caused by an impaired triglyceride synthesis, a reduced fatty acid β-oxidation, or both. Patients with steatosis can progress to non-alcoholic steatohepatitis (NASH), a more severe form of NAFLD. NASH involves hepatocellular injury and liver inflammation [4]. The majority of individuals with NAFLD are asymptomatic. In some patients, NAFLD is diagnosed after a blood test, which shows elevated levels of liver enzymes (alanine transaminase, aspartate aminotransferase, and alkaline phosphatase) or through an ultrasound scan. Individuals who are either diabetic, obese, or suffer from metabolic syndrome will be suspected to have NAFLD [5].

Management of body weight through increased physical activity and improvement in diet can help in managing NAFLD and delaying disease progression. However, lifestyle interventions may not be effective in many cases [6,7,8]. Pharmacological treatment for NAFLD patients includes the use of insulin-sensitizing agents (thiazolidinediones (TZDs)). However, clinical studies could not demonstrate the effectiveness of these drugs in combined treatment of NAFLD with metformin [9,10].

The functions of Kupffer cells include clearance of pathogens, immune complexes, endotoxins, cellular debris, tumor cells, and the maintenance of immunological tolerance at steady state condition [11,12,13]. Kupffer cells are also involved in insulin resistance and fatty liver disease [14]. There are two subsets of Kupffer cells: the cytokine-producing CD11b^+^ cells and the phagocytic and ROS-producing CD68^+^ cells [15]. PPARβ/δ expression in adult mouse liver was found to be at moderate to high levels [16]. In the liver of rat on a chow diet, the expression of *Pparb/d* was highest in sinusoidal endothelial cells (LSECs). The next higher *Pparb/d* expression is in hepatocytes and liver resident macrophages (Kupffer cells) and the impact of PPARβ/δ on hepatic lipid metabolism has been shown to involve these cells [17].

PPARβ/δ plays a central role in fatty acid oxidation and improves lipid and cholesterol profiles, which prevents obesity [18,19]. PPARβ/δ is also involved in regulating the alternative activation of Kupffer cells. Under IL4 and IL13 stimulation, Kupffer cell activation to the macrophage M2, which has anti-inflammatory activity, requires PPARβ/δ. Lower insulin sensitivity and oxidative metabolism were observed in hematopoietic *Pparb/d* deficient obese mice, which presents impaired alternative activation of Kupffer cells [20]. Although PPARβ/δ demonstrates an anti-inflammatory effect in preventing cancer before its development, activation of PPARβ/δ after the development of cancer can promote angiogenesis and cancer growth [21]. PPARβ/δ is also involved in chronic inflammation in the colon and colitis-associated carcinogenesis [22,23].

Fibroblast-specific protein 1 (FSP1) belongs to the S100 superfamily of cytoplasmic calcium-binding proteins. It is also known as S100A4. S100 proteins do not have enzymatic activity upon the formation of homo- or hetero-dimers. However, they can regulate the function of other proteins by binding to them [24]. Studies have demonstrated that FSP1 is expressed in fibroblasts in various organs undergoing tissue remodeling, which include lung, kidney, and heart [25,26,27].

In addition, there is high FSP1 expression in adult mouse and rat tissues, including spleen, thymus, bone marrow, absorptive and keratinized epithelia, and in T-lymphocytes, neutrophils, and macrophages [28,29,30]. An increase in FSP1-positive cells was also observed in both mouse experimental liver injury and liver injury in patients [31]. A recent study showed that a subpopulation of macrophages secretes FSP1 during liver fibrosis [32].

We derived a new mouse line from crossing *Pparb/d^fl/fl^* mice with FSP1-cre mice. The skin and gut phenotypes of the FSP1cre-*Pparb/d^−/−^* mouse were previously characterized [33,34]. The present study explores the effects of *Pparb/d* deletion in FSP1-expressing hepatic non-parenchymal cells on the liver. We hypothesized that the deletion of *Pparb/d* in FSP1-expressing non-parenchymal cells in the liver would have an impact on liver metabolism and homeostasis, particularly involving lipid metabolism and possibly also steatosis.

## 2. Results

### 2.1. Deletion of Pparb/d in FSP1^+^CD11b^+^ Cells

**Figure 1:**
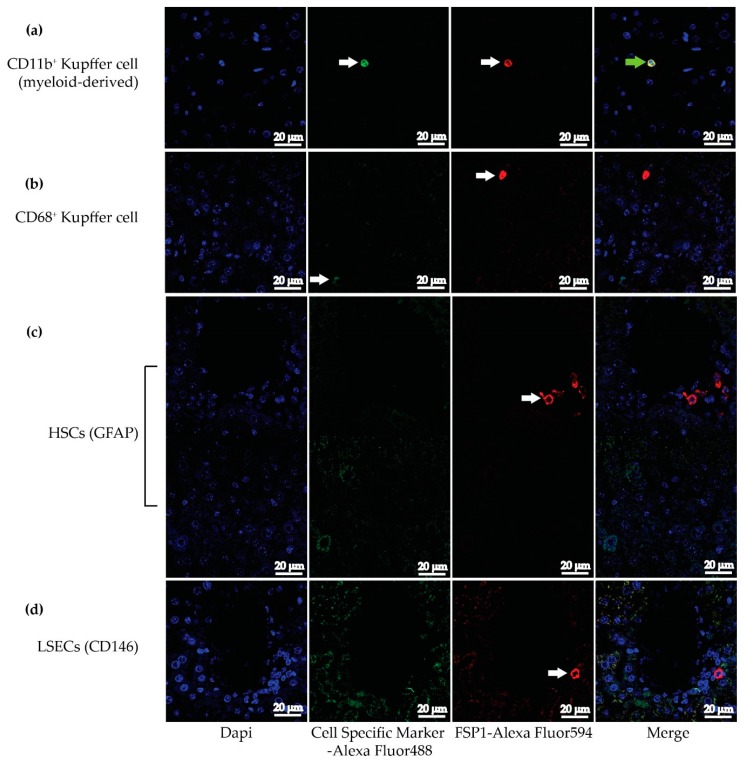
Double immunofluorescence staining in Fibroblast-specific protein 1 (FSP1)cre-*Pparb/d^−/−^* liver for Glial fibrillary acidic protein (GFAP), CD146, CD68 or CD11b with FSP1. (**a**) CD11b-expressing liver resident macrophages express FSP1. (**b**) CD68-expressing Kupffer cells do not express FSP1. (**c**) GFAP-expressing hepatic stellate cells (HSCs) do not express FSP1. (**d**) CD146-expressing LSECs do not express FSP1. Arrows indicate staining; the green arrow indicates positive co-staining of CD11b and FSP1.

Mouse hepatocytes do not express FSP1 (not shown). Thus, we aimed to identify FSP1-expressing non-parenchymal cell populations in the liver. We conducted double immunofluorescence co-staining of FSP1 with other liver cell-type markers. CD11b is a subunit protein of complement receptor 3, which is expressed in macrophages. Both monocytes and macrophages expressed CD68, which is a glycoprotein that binds to low-density lipoprotein. Resident liver macrophages belong to CD11b^+^CD68^−^ and CD11b^−^CD68^+^ cell populations [15]. Hence, anti-CD11b and anti-CD68 were used to identify CD11b^+^CD68^−^ and CD11b^−^CD68^+^ resident liver macrophage populations, respectively. We observed co-staining of FSP1 and CD11b identifying CD11b^+^ cells as FSP1 expressing cells (Figure 1a). There was no FSP1 staining in CD68^+^ cells (Figure 1b). Hence, this observation suggests that FSP1 is expressed in resident liver macrophages, particularly in CD11b^+^CD68^−^ resident liver macrophages.

Other non-parenchymal hepatic cells were also investigated for co-expression of specific markers and FSP1. Glial fibrillary acidic protein (GFAP) is an intermediate filament protein expressed in numerous central nervous system cells, including astrocytes. GFAP is also found to be a specific biomarker of a hepatic stellate cell (HSC). We observed no co-staining of FSP1 and GFAP in HSCs (Figure 1c), suggesting that HSCs do not express FSP1.

The LSECs were identified using a specific biomarker, CD146. CD146 is a transmembrane glycoprotein which is a member of the IgG superfamily of cell adhesion molecules. We found that there was no FSP1 co-staining with CD146-positive LSECs (Figure 1d), indicating that LSECs do not express FSP1.

**Figure 2:**
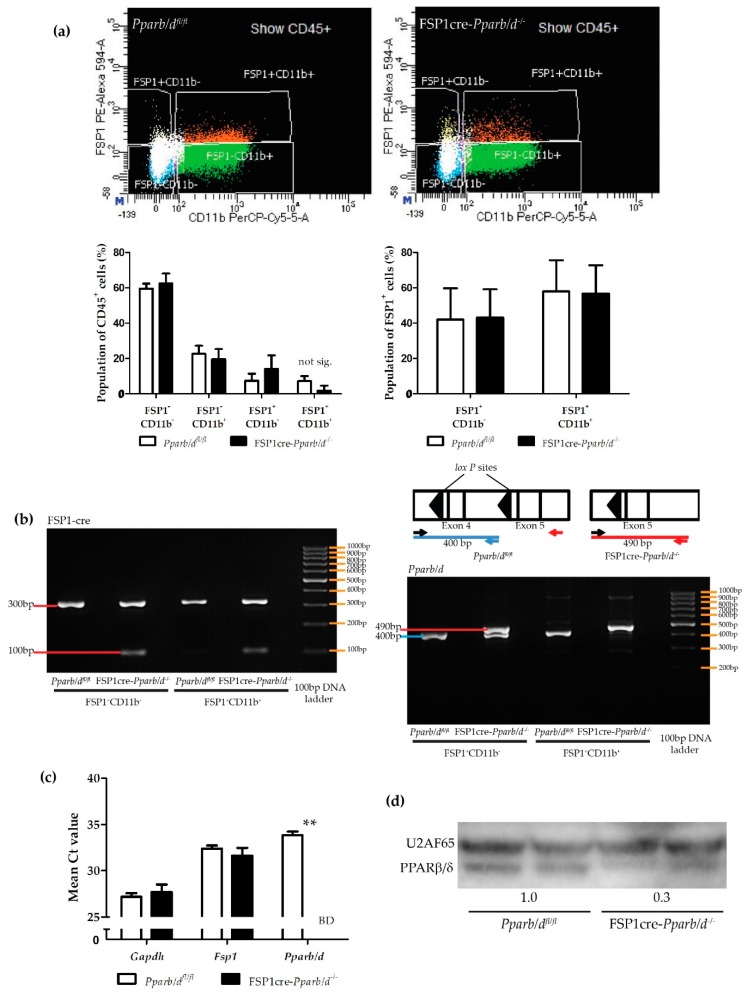
Distribution of FSP1^+^CD11b^+^ and FSP1^+^CD11b^−^ cells in non-parenchymal liver cells and genotyping of these isolated cells. Majority of the FSP1^+^ cells express CD11b. (**a**) *Pparb/d^fl/fl^* and FSP1cre-*Pparb/d^−/−^* liver FACS plots showing the CD45^+^ cell population, which is stained for FSP1 and CD11b, and graphs showing the distribution of cells from each quadrant in percentage (data presented as mean ± s.e.m; *n* = 6 biological replicates per group). Two-tailed Mann-Whitney test of the data did not find significant differences between the FSP1cre-*Pparb/d^−/−^* and *Pparb/d^fl/fl^* genotypes. (**b**) Schematic diagram showing the relative position of PCR genotyping primers for the *Pparb/d* lox/lox (400 bp band) and the deleted *Pparb/d* exon 4 alleles (490 bp band). FSP1-cre genotyping: 100 bp band indicates FSP1-cre. 300 bp band is from the endogenous *Fsp1* gene. In both gels, the rightmost lane depicts the molecular weight 100 bp ladder. (**c**) *Fsp1* and *Pparb/d* gene expression in isolated FSP1^+^CD11b^+^ cells from P2 *Pparb/d^fl/fl^* and FSP1cre-*Pparb/d^−/−^* livers. BD, below detection. Two-tailed Mann-Whitney test of the data shown as mean ± s.e.m, *n* = 5 biological replicates per group. ** *P* < 0.01; FSP1cre-*Pparb/d^−/−^* vs. *Pparb/d^fl/fl^* controls. (**d**) Immunoblot analysis of *PPARβ/δ* in P2 *Pparb/d^fl/fl^* and FSP1cre-*Pparb/d^−/−^* whole livers. Representative blots from 2 livers for each genotype showing lower PPARβ/δ expression in FSP1cre-*Pparb/d^−/−^* livers are shown. The mean PPARβ/δ expression level in *Pparb/d ^fl/fl^* livers was arbitrarily given a value of 1. This level is more than 3 times lower in FSP1cre-*Pparb/d^−/−^* livers. The full-size western blot is shown in Figure SM2a. U2AF65 is used as loading and transfer control.

We validated the co-expression of CD11b and FSP1 in non-parenchymal liver cells using flow cytometry (Figure 2a). All leucocytes express CD45, which is a receptor-linked protein tyrosine phosphatase [35]. Hence, CD45 is commonly used as a marker of bone marrow-derived cells. About 14.5% of CD45-expressing liver-derived cells expressed FSP1. About 57% of these FSP1-expressing cells were CD11b^+^ cells. Altogether, our results showed that non-parenchymal hepatic cells, such as HSCs, LSECs, and CD68^+^ Kupffer cells, do not express FSP1. We concluded that CD11b^+^ resident liver macrophages express FSP1.

Using flow cytometry cell sorting, we isolated FSP1^+^CD11b^+^ cells from both *Pparb/d^fl/fl^* and FSP1cre-*Pparb/d^−/−^* livers. *Pparb/d* genotyping showed a total deletion in the targeted exon 4 of *Pparb/d*, indicated by the 490 bp band, in FSP1^+^CD11b^+^ cells isolated from FSP1cre-*Pparb/d^−/−^* liver. As expected, only the 400 bp *Pparb/d* lox/lox band was present in FSP1^+^CD11b^+^ cells from *Pparb/d^fl/fl^* liver (Figure 2b). Furthermore, genotyping of these cells by PCR analysis showed that the expected FSP1-cre band (100 bp) was present in the FSP1^+^CD11b^+^ cells isolated from FSP1cre-*Pparb/d^−/−^* liver but not from *Pparb/d^fl/fl^* liver. The FSP1 endogenous control band (300 bp) was present in both FSP1^+^CD11b^−^ and FSP1^+^CD11b^+^ cells isolated from *Pparb/d^fl/fl^* and FSP1cre-*Pparb/d^−/−^* livers.

Remarkably, the level of PPARβ/δ mRNA was below detection in isolated FSP1^+^CD11b^+^ cells from P2 FSP1cre-*Pparb/d^−/−^* livers (Figure 2c). As a consequence of the invalidation of *Pparb/d*, the level of PPARβ/δ protein in whole liver extracts was lower in FSP1cre-*Pparb/d^−/−^* livers compared to *Pparb/d^fl/fl^* livers (Figure 2d). Due to the very low abundance of FSP1^+^CD11b^+^ cells, it was technically not possible to determine the PPARβ/δ protein level in the lysates from these cells by using western blot.

### 2.2. More and Larger Intracellular Lipid Droplets in FSP1cre-Pparb/d^−/−^ Livers at P2 and Week 4

**Figure 3:**
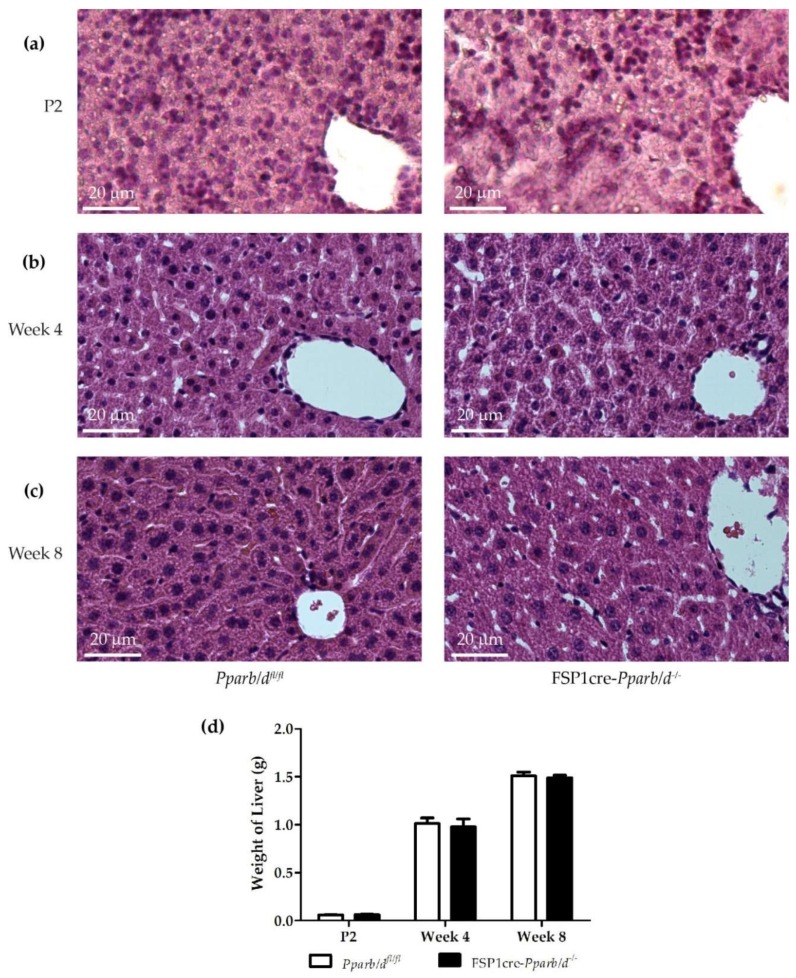
H&E staining of liver sections and comparison of the liver weight in *Pparb/d^fl/fl^* and FSP1cre-*Pparb/d^−/−^* mice at three age points. (**a**) Postnatal day 2 (P2) livers. (**b**) Week 4 of age livers. (**c**) Week 8 of age livers. (**d**) Weight of livers of the two genotypes at each age is shown. Data are shown as mean ± s.e.m, *n* = 10–14 biological replicates per group.

To define the potential role of PPARβ/δ in FSP1-expressing non-parenchymal hepatic cells on liver structure and function, we analyzed the livers of pups and young animals at time points of important metabolic changes (suckling—P2; weaning—week 4; transition to adulthood—week 8). We examined the liver from FSP1cre-*Pparb/d^−/−^* mice at postnatal day 2 (P2), week 4, and week 8 of age after hematoxylin and eosin staining. Livers from *Pparb/d^fl/fl^* mice at the same age served as controls for comparison. FSP1cre-*Pparb/d^−/−^* livers appeared less compacted when compared with their *Pparb/d^fl/fl^* counterparts because of extended sinusoids. Furthermore, the hepatocytes in FSP1cre-*Pparb/d^−/−^* mice presented a more pronounced microvesicular structure, likely indicative of more intracellular lipid droplets (Figure 3a,b). At week 8 of age, the compactness of the hepatic cells in *Pparb/d^fl/fl^* and FSP1cre-*Pparb/d^−/−^* livers was similar and some microvesicles remained in the livers of both FSP1cre-*Pparb/d^−/−^* and *Pparb/d^fl/fl^* mice (Figure 3c). There was no significant difference in the weight of the livers of both genotypes (Figure 3d).

**Figure 4:**
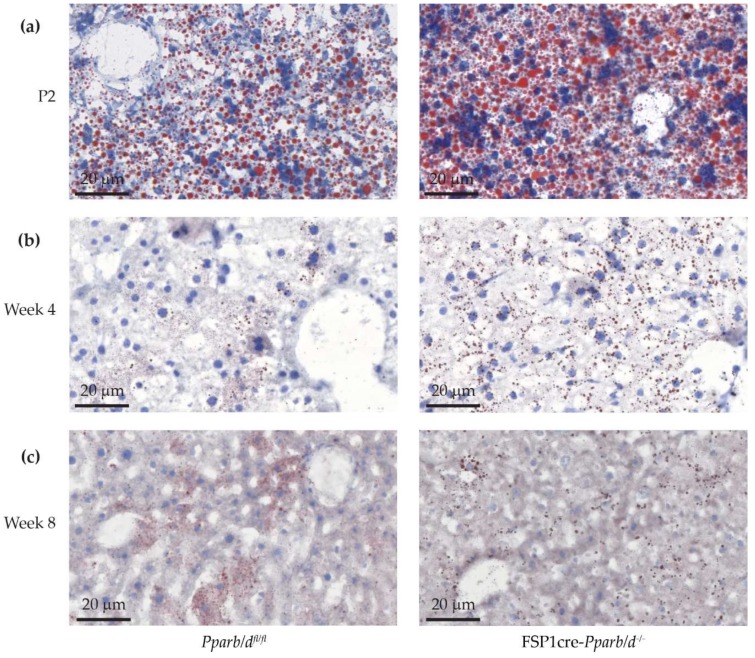
Oil Red O staining of liver sections from *Pparb/d^fl/fl^* and FSP1cre-*Pparb/d^−/−^* mice at three age points. Larger and more lipid droplets are observed at P2 livers in FSP1cre-*Pparb/d^−/−^* mice. There are also more droplets at week 4. (**a**) P2 livers. (**b**) Week 4 livers. (**c**) Week 8 livers.

Because the hematoxylin and eosin staining of liver sections in FSP1cre-*Pparb/d^−/−^* mice revealed a more pronounced microvesicular like structure than in the liver of *Pparb/d^fl/fl^* mice, we performed Oil red O staining to detect intracellular lipid droplets. As expected under the milk diet, which is high in fat content, lipid droplets were present in P2 hepatocytes of both *Pparb/d^fl/fl^* and FSP1cre-*Pparb/d^−/−^* mice. However, the droplets were more abundant and larger in the FSP1cre-*Pparb/d^−/−^* livers (Figure 4a). At week 4 in both *Pparb/d^fl/fl^* and FSP1cre-*Pparb/d^−/−^* livers, the lipid droplets were more abundant in hepatocytes adjacent to the hepatic sinusoids, and the number of droplets was higher in hepatocytes of the FSP1cre-*Pparb/d^−/−^* mice. Furthermore, at week 4, the droplets are much smaller than at P2 (Figure 4b).

Remarkably, at week 8 of age, the number of the lipid droplets appeared similar in the livers of both *Pparb/d^fl/fl^* and FSP1cre-*Pparb/d^−/−^* mice (Figure 4c). Again, the lipid droplets were preferentially localized within hepatocytes that were adjacent to the hepatic sinusoids. Together, our results indicate that the observed phenotype (higher number and larger size of lipid droplets) only occurs in livers of the FSP1cre-*Pparb/d^−/−^* during early life (at P2 and to a lesser extent at week 4).

### 2.3. Higher Triglyceride Levels in the Liver of FSP1cre-Pparb/d^−/−^ Mice at P2 and Week 4

The observed lipid droplet phenotype prompted us to quantify it. To this end, we measured lipid levels in both genotypes at the 3 age points. We hypothesized that the FSP1cre-*Pparb/d^−/−^* livers would have higher levels of total lipids, especially at P2 and week 4. The liver lipids were extracted using the Cell Biolabs lipid extraction kit and measured using the Cell Biolabs total lipids quantification kit. The readings were normalized to the protein concentration in the respective samples (µg lipids per µg protein).

**Figure 5:**
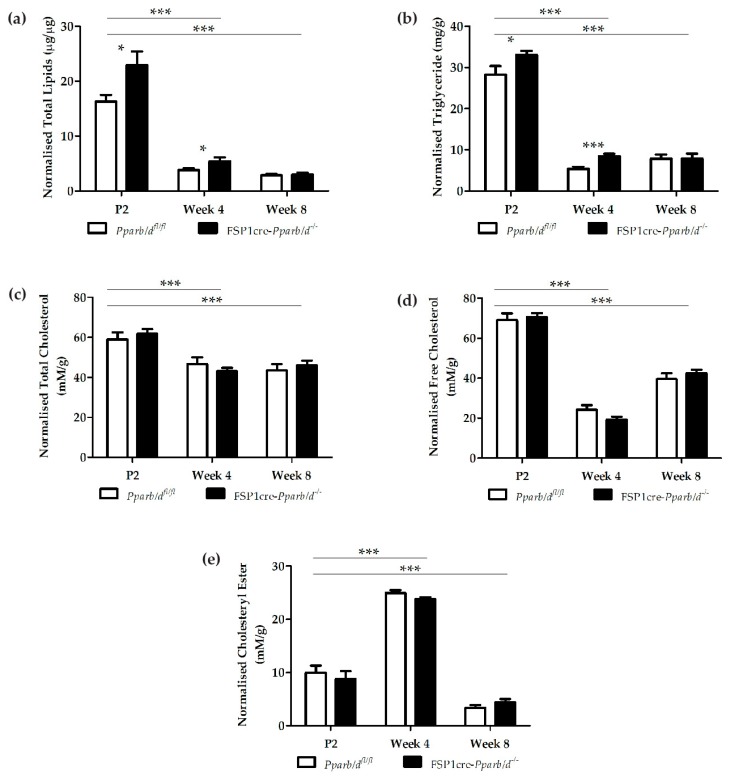
Lipid profiling of *Pparb/d^fl/fl^* and FSP1cre-*Pparb/d^−/−^* livers at the three age points. (**a**) Normalized total lipids. (**b**) Normalized triglycerides. (**c**) Normalized total cholesterol. (**d**) Normalized free cholesterol. (**e**) Normalized cholesteryl ester. Two-tailed Mann-Whitney test was used to compare differences between *Pparb/d^fl/fl^* and FSP1cre-*Pparb/d^−/−^* livers of the same age. Two-way ANOVA was used to compare the differences between the ages of *Pparb/d^fl/fl^* and FSP1cre-*Pparb/d^−/−^* livers. Data are shown as mean ± s.e.m, *n* = 9 to 10 biological replicates per group. * *P* < 0.05, *** *P* < 0.001.

As expected from the histological observations, the level of total lipids was higher in P2 and week 4 livers of FSP1cre-*Pparb/d^−/−^* mice. In P2 pups, there was more than a 40.4% increase in total lipids in livers of FSP1cre-*Pparb/d^−/−^* compared to *Pparb/d^fl/fl^* mice (FSP1cre-*Pparb/d^−/−^* = 22.90 ± 8.00 μg/μg vs. *Pparb/d^fl/fl^* = 16.31 ± 3.74 μg/μg; *P* < 0.05) (Figure 5a). Similarly, higher lipid levels in FSP1cre-*Pparb/d^−/−^* livers were observed at week 4 (FSP1cre-*Pparb/d^−/−^* = 5.45 ± 2.11 μg/μg vs. *Pparb/d^fl/fl^* = 3.81 ± 0.97 μg/μg; *P* < 0.05). However, as expected from the histological observations, the levels were lower in week 4 compared to P2. Interestingly, the total lipid levels between the livers of the two genotypes were similar at week 8 (FSP1cre-*Pparb/d^−/−^* = 3.01 ± 1.09 μg/μg vs. *Pparb/d^fl/fl^* = 2.88 ± 0.73 μg/μg) (Figure 5a). Collectively, these results show that the total lipid levels in FSP1cre-*Pparb/d^−/−^* livers were significantly higher than in *Pparb/d^fl/fl^* livers at P2 and week 4, with a significant decrease between P2 (suckling) and week 4 (post-weaning).

Next, we measured the levels of triglycerides in these livers. Stored triglycerides serve as an energy source and play a key role in metabolism. Excess carbohydrates and fats that are not immediately used by the body are converted to triglycerides. Hence, we hypothesized that the level of triglycerides might be different in the livers of FSP1cre-*Pparb/d^−/−^* and *Pparb/d^fl/fl^* mice. The quantification of triglycerides was done with the Cell Biolabs triglyceride quantification kit. The readings were normalized to the total protein in the respective samples (mg triglycerides to g protein).

Interestingly, the level of triglycerides in livers of FSP1cre-*Pparb/d^−/−^* mice at P2 and week 4 were significantly higher than in livers of *Pparb/d^fl/fl^* mice (P2: FSP1cre-*Pparb/d^−/−^* = 33.09 ± 2.87 mg/g vs. *Pparb/d^fl/fl^* = 28.24 ± 6.64 mg/g; *P* < 0.05; week 4: FSP1cre-*Pparb/d^−/−^* = 8.50 ± 1.79 mg/g vs. *Pparb/d^fl/fl^* = 5.38 ± 1.76 mg/g; *P* < 0.01). However, at week 8, the levels of triglycerides were similar in both genotypes (FSP1cre-*Pparb/d^−/−^* = 7.89 ± 3.87 mg/g vs. *Pparb/d^fl/fl^* = 7.84 ± 3.29 mg/g) (Figure 5b).

In addition to changes in triglyceride levels, we hypothesized that cholesterol levels might be changed as well. Hence, we quantified total cholesterol, free cholesterol, and cholesteryl ester using the Cell Biolabs total cholesterol kit. The readings were normalized to the weight of the respective tissue samples. Surprisingly, there was no difference in total cholesterol content between livers of FSP1cre-*Pparb/d^−/−^* and *Pparb/d^fl/fl^* mice at all three ages (Figure 5c). However, total cholesterol was significantly lower in week 4 compared to P2 by at least 20% (P2: FSP1cre-*Pparb/d^−/−^* = 61.83 ± 7.56 mM/g and *Pparb/d^fl/fl^* = 59.06 ± 10.97 mM/g; Week 4: FSP1cre-*Pparb/d^−/−^* = 43.05 ± 5.44 mM/g and *Pparb/d^fl/fl^* = 46.74 ± 10.71 µM/mg). The level of total cholesterol was similar in both genotypes at week 8 (Week 8: FSP1cre-*Pparb/d^−/−^* = 46.17 ± 7.05 mM/g and *Pparb/d^fl/fl^* = 43.56 ± 9.68 mM/g).

Moreover, there was no significant difference in free cholesterol across all three age points between FSP1cre-*Pparb/d^−/−^* and *Pparb/d^fl/fl^* livers (Figure 5d). The free cholesterol level was significantly lower at week 4 compared to P2 by at least 60% (P2: FSP1cre-*Pparb/d^−/−^* = 70.65 ± 6.05 mM/g and *Pparb/d^fl/fl^* = 69.04 ± 10.74 mM/g and, Week 4; FSP1cre-*Pparb/d^−/−^* = 19.27 ± 4.60 mM/g and *Pparb/d^fl/fl^* = 24.90 ± 6.77 mM/g) and significantly higher by at least 60% at week 8 compared to week 4 (Week 8: FSP1cre-*Pparb/d^−/−^* = 42.58 ± 5.28 mM/g and *Pparb/d^fl/fl^* = 39.55 ± 9.03 mM/g).

Since the free cholesterol and total cholesterol levels were not different between the livers of FSP1cre-*Pparb/d^−/−^* and *Pparb/d^fl/fl^* mice, we expected the level of cholesteryl ester to also be similar between the two genotypes. Indeed, this expectation was confirmed (Figure 5e). The cholesteryl ester level was significantly higher at week 4 compared to P2 by ~50% (P2: FSP1cre-*Pparb/d^−/−^* = 8.82 ± 4.60 mM/g and *Pparb/d^fl/fl^* = 9.98 ± 4.21 mM/g, Week 4: FSP1cre-*Pparb/d^−/−^* = 23.79 ± 1.27 mM/g and *Pparb/d^fl/fl^* = 24.93 ± 1.72 mM/g), but lower by ~80% at week 8 (Week 8: FSP1cre-*Pparb/d^−/−^* = 4.47 ± 1.71 mM/g and *Pparb/d^fl/fl^* = 3.39 ± 1.50 mM/g). Altogether, there was no significant difference in total cholesterol, free cholesterol, and cholesteryl ester between the livers of FSP1cre-*Pparb/d^−/−^* and *Pparb/d^fl/fl^* mice across all three age points.

### 2.4. Upregulation of Genes Involved in Fatty Acid β-Oxidation, Fatty Acid Synthesis and Triglyceride Synthesis in FSP1cre-Pparb/d^−/−^ Liver at P2 and Week 4

Based on the important differences in triglyceride levels between the livers of FSP1cre-*Pparb/d^−/−^* and *Pparb/d^fl/fl^* mice, we examined the expression of genes involved in fatty acid β-oxidation and triglyceride synthesis. As PPARs (PPARα, PPARβ/δ, and PPARγ) are lipid-activated transcription factors that play an important role in energy metabolism, we measured their expression levels in the liver of both genotypes. We were interested in determining whether changes in PPAR isotype expression could be leading to the increased lipid accumulation and elevated triglyceride levels in the livers of P2 and week 4 FSP1cre-*Pparb/d^−/−^* mice.

**Figure 6:**
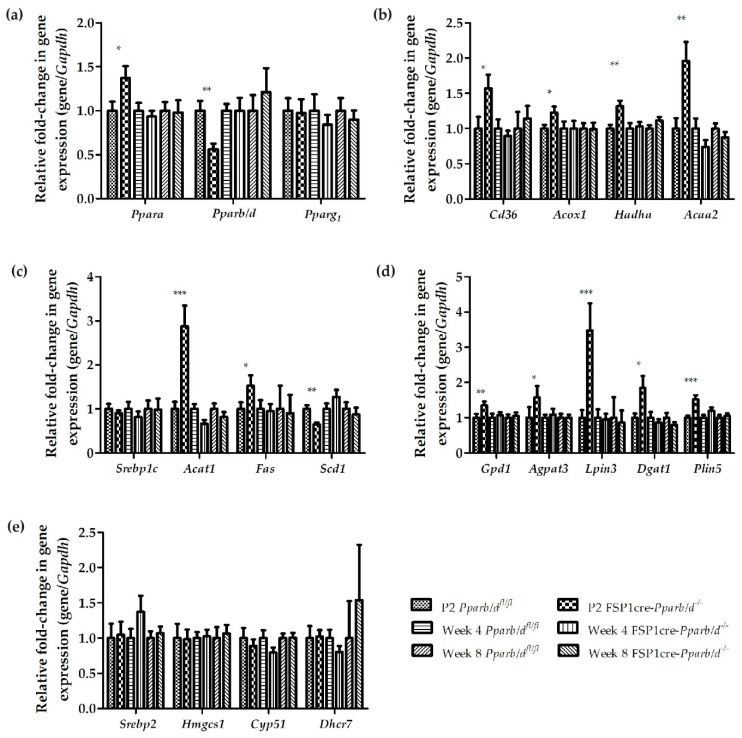
Effect of *Pparb/d* deletion on liver gene expression at P2, week 4, and week 8. Relative fold change in the mRNA levels of specific genes in livers of *Pparb/d^fl/fl^* and FSP1cre-*Pparb/d^−/−^* mice as determined by real-time qPCR. (**a**) Expression of *Ppara, Pparb/d, and Pparg*. (**b**) Fatty acid β-oxidation genes. (**c**) Fatty acid synthesis genes. (**d**) Triglyceride synthesis genes and *Plin5*. (**e**) Cholesterol synthesis genes. Values are normalized to the expression of *Gapdh*. Normalized values from controls were arbitrarily assigned a value of 1. Two-tailed Mann-Whitney test with values shown as mean ± s.e.m, *n* = 10 biological replicates per group. * *P* < 0.05, ** *P* < 0.01, *** *P* < 0.001; FSP1cre-*Pparb/d^−/−^* vs. *Pparb/d^fl/fl^* controls.

Remarkably, the expression of PPARα was upregulated by 31.1% in P2 FSP1cre-*Pparb/d^−/−^* livers. PPARα is involved in the regulation of lipid metabolism, such as lipogenesis and fatty acid oxidation (Figure 6a). However, expression of *Ppara* in the livers of *Pparb/d^fl/fl^* and FSP1cre-*Pparb/d^−/−^* mice was not different at weeks 4 and 8. There was no significant change in *Pparg_1_* expression between the two genotypes. However, there was a reduced expression of *Pparb/d* by 43.8% in the liver of P2 FSP1cre-*Pparb/d^−/−^* mice. The expression of *Pparb/d* between livers of *Pparb/d^fl/fl^* and FSP1cre-*Pparb/d^−/−^* mice at weeks 4 and 8 was similar.

We reported above that there was a higher number of lipid droplets and total lipid levels in the liver of P2 and week 4 FSP1cre-*Pparb/d^−/−^* mice. First, this prompted us to examine the expression level of genes involved in fatty acid β-oxidation. Although there was higher lipid accumulation in P2 FSP1cre-*Pparb/d^−/−^* livers compared to *Pparb/d^fl/fl^* livers, fatty acid β-oxidation genes such as *Cd36*, *acyl-CoA oxidase 1* (*Acox1*), *Hadha*, and *Acaa2* were all upregulated in P2 FSP1cre-*Pparb/d^−/−^* livers (Figure 6b). CD36, which functions as a fatty acid transporter, had its gene expression upregulated by 57.2%. *Acox1* is the first enzyme in the fatty acid β-oxidation pathway, which catalyzes the desaturation of acyl-CoAs to 2-trans-enoyl-CoAs. *Acox1* expression was increased by 22.5%. *Hadha*, which catalyzes the mitochondrial beta-oxidation of long-chain fatty acids to β-ketoacyl-CoA, had its gene expression upregulated by 31.7%. The expression of the gene coding for *Acaa2*, which catalyzes the final step of the mitochondrial fatty acid beta-oxidation, was increased by 95.9%. No significant change in the expression of these fatty acid β-oxidation genes was observed in week 4 and week 8 livers of *Pparb/d^fl/fl^* and FSP1cre-*Pparb/d^−/−^* mice.

Accumulation of lipid can result from an increase in fatty acid synthesis. Therefore, we examined genes expressed in the fatty acid synthesis pathway, such as *Acat1*, *Fas*, and *Scd1* (Figure 6c). The most significantly upregulated gene was *Acat1*. ACAT1 is a mitochondria-localized enzyme that catalyzes the reversible formation of acetoacetyl-CoA from two molecules of acetyl-CoA to initiate fatty acid synthesis. We observed that the expression of *Acat1* was increased by 187.7%, while that of *Fas* was increased by 52.8%. SCD1 is an enzyme involved primarily in oleic acid synthesis. Unexpectedly, *Scd1* expression was downregulated by about 35.3% in P2 FSP1cre-*Pparb/d^−/−^* livers. In contrast, there was no significant change in the expression of these fatty acid synthesis genes between the two genotypes in week 4 and week 8 livers. Expression of *Srebp1c*, which codes for a transcription factor that regulates genes required for glucose metabolism and fatty acid and lipid production, remained similar at the 3 age points in both genotypes.

The expression of genes involved in the triglyceride synthesis pathway or lipogenesis, such as *Gpd1*, 1-acylglycerol-3-phosphate O-acyltransferase 3 (*Agpat3*), *Lpin3*, and *Dgat1*, was also examined (Figure 6d). In parallel with the upregulation of genes in the fatty acid synthesis pathway in P2 FSP1cre-*Pparb/d^−/−^* livers, the expression of these four triglyceride synthesis genes was also increased. GPD1, along with GPD2, plays a critical role in lipid metabolism by transferring glycerol-3-phosphate from the cytosol to mitochondria. This process takes place after catalyzing the conversion of NADH to NAD+ and glycerol-3-phosphate. *Gpd1* expression was increased by 35.3% in P2 FSP1cre-*Pparb/d^−/−^* compared to *Pparb/d^fl/fl^* livers. AGPAT3 is a mitochondrial acyltransferase that converts lysophosphatidic acid into phosphatidic acid in the triglyceride synthesis pathway. The expression level of *Agpat3* was upregulated by 57.5% in P2 FSP1cre-*Pparb/d^−/−^* livers. LPIN3 catalyzes the dephosphorylation of phosphatidic acid to form diacylglycerol. Diacylglycerol is the precursor of both triglycerides and phospholipids. A staggering 248.3% increase in *Lpin3* expression was observed in P2 FSP1cre-*Pparb/d^−/−^* livers. In the final step of triglyceride synthesis, DGAT1 catalyzes the conversion of diacylglycerol and fatty acyl CoA to triacylglycerol. *Dgat1* expression was elevated by 84.9% in P2 FSP1cre-*Pparb/d^−/−^* livers. Lastly, *Plin5*, which encodes an intracellular lipid droplet coating protein promoting triglyceride stores and preventing them from lipolytic degradation, was increased by 52.1% in P2 FSP1cre-*Pparb/d^−/−^* livers. In contrast to P2 livers, there were no significant changes in the expression of all these genes between both genotypes in week 4 and 8 livers. Collectively, these qPCR results demonstrated that both fatty acid and triglyceride synthesis pathways were upregulated in the livers of FSP1cre-*Pparb/d^−/−^* mice, which is consistent with the lipid droplets accumulation and higher total lipid levels in these livers. It is noteworthy that in parallel fatty acid β-oxidation genes were upregulated as well.

As mentioned above, there was no difference in total cholesterol, free cholesterol, and cholesteryl ester in the livers of *Pparb/d^fl/fl^* and FSP1cre-*Pparb/d^−/−^* mice at P2, week 4, and week 8. Hence, we conducted a gene expression analysis on cholesterol synthesis genes to consolidate (or not consolidate) these results. *Srebp2*, *Hmgcs1*, cytochrome P450 family 51 subfamily A member 1 (*Cyp51*), and *Dhcr7* expressions were found to be similar in both liver genotypes at all 3-time points (Figure 6e). Hence, these results further suggest that the accumulation of lipid droplets and a higher level of total lipids in P2 and week 4 FSP1cre-*Pparb/d^−/−^* livers was not the result of increased cholesterol synthesis.

### 2.5. Increase in Hadha, Phospho-ACLY, Phospho-ACC and GPD2 in the Liver of P2 FSP1cre-Pparb/d^−/−^ Mice

P2 livers of FSP1cre-*Pparb/d^−/−^* mice exhibited more lipid droplets and higher levels of total lipids and triglycerides. In parallel, the expression of genes involved in lipid and triglyceride synthesis was also increased, as well as genes involved in fatty acid oxidation. We complemented these results by analyzing the expression and phosphorylation level of some lipid metabolism proteins in P2 livers of FSP1cre-*Pparb/d^−/−^* compared to control *Pparb/d^fl/fl^* mice.

**Figure 7:**
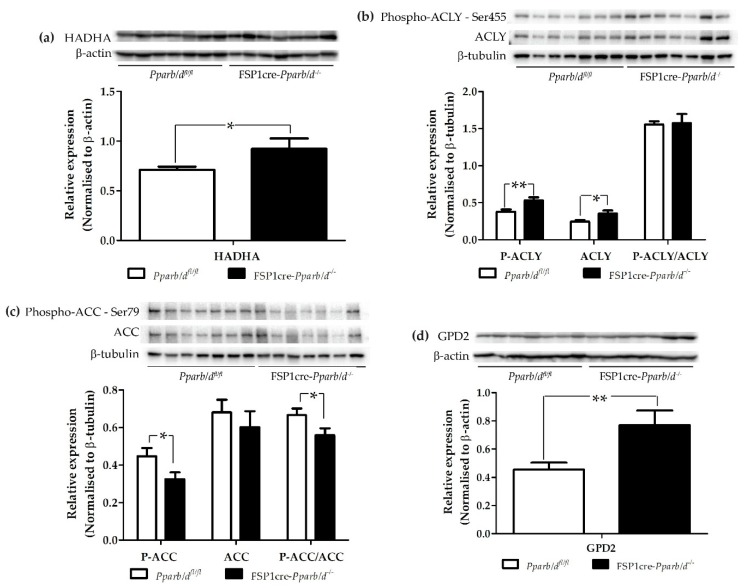
Expression levels of proteins in fatty acid β-oxidation, fatty acid synthesis, and triglyceride synthesis pathways. Immunoblot analysis of indicated proteins in the liver of P2 *Pparb/d^fl/fl^* and FSP1cre-*Pparb/d^−/−^* mice. Representative blots from 7 mice (biological replicates) for each genotype and the results of the 6-7 mice (biological replicates) are shown in bar graphs. Two-tailed Mann-Whitney test with values shown as mean ± s.e.m. * *P* < 0.05, ** *P* < 0.01; FSP1cre-*Pparb/d^−/−^* vs. *Pparb/d^fl/fl^* controls. (**a**) HADHA. β-actin was used as loading and transfer control. (**b**) Phospho-ACLY and ACLY. β-tubulin was used as loading and transfer control. (**c**) Phospho-ACC and ACC. β-tubulin was used as loading and transfer control. (**d**) GPD2. β-actin was used as loading and transfer control. The full size original western blots are shown in Figure SM2.

First, we observed that the trifunctional HADHA protein involved in fatty acid β-oxidation was upregulated by 30.0% in the liver of FSP1cre-*Pparb/d^−/−^* mice (Figure 7a). Hence, this result is in line with the upregulated HADHA mRNA level in the liver of FSP1cre-*Pparb/d^−/−^* mice (Figure 6b).

Second, we tested ATP citrate lyase (ACLY) which cleaves citrate into oxaloacetate and acetyl-CoA, the latter serving as a substrate for cholesterol and fatty acid synthesis. Phosphorylation of ACLY at Ser455 inhibits the homotopic allosteric regulation through citrate, which promotes the hydrolysis process to increase the yield of acetyl-CoA. Hence, ACLY has a central role in triglyceride synthesis. Both ACLY and phospho-ACLY were significantly upregulated in the liver of FSP1cre-*Pparb/d^−/−^* mice by 40.0% and 43.7%, respectively (Figure 7b). Hence, we speculate that there is more acetyl-CoA in the liver of FSP1cre-*Pparb/d^−/−^* mice available for fatty acid synthesis.

Third, we measured acetyl-CoA carboxylase (ACC), which plays a crucial role in the fatty acid synthesis pathway. In the biogenesis of long-chain fatty acids, the carboxylation process of acetyl-CoA to malonyl-CoA is catalyzed by ACC. Phosphorylation at Ser79 in ACC by AMP-activated protein kinase (AMPK) reduces the enzymatic activity of ACC. The protein expression level of ACC was similar in the livers of *Pparb/d^fl/fl^* and FSP1cre-*Pparb/d^−/−^* mice (Figure 7c). However, phospho-ACC was significantly downregulated by 27.4% in the liver of FSP1cre-*Pparb/d^−/−^* mice, which should result in a more active enzyme in these mice compared to the control mice. These results, together with those from ACLY, suggest an upregulation of fatty acid synthesis in the liver of FSP1cre-*Pparb/δ^−/−^* mice.

Last, we examined GPD2, which along with GPD1, plays a critical role in triglyceride synthesis by transferring glycerol-3-phosphate from the cytosol to mitochondria. This process takes place after catalyzing the conversion of NADH to NAD+ and glycerol-3-phosphate. We observed an upregulation of GPD2 expression by 68.6% in the liver of FSP1cre-*Pparb/d^−/−^* mice (Figure 7d). Hence, this result further suggests an upregulation of triglyceride synthesis in the liver of FSP1cre-*Pparb/d^−/−^* mice. In contrast to ACLY, ACC, and GPD2, only the mRNA level of HADHA was not upregulated in P2 FSP1cre-*Pparb/d^−/−^* livers compared with *Pparb/d^fl/fl^* livers (Figure 6b, Figure SM1). This observation suggests a post-transcriptional regulation of these three genes in the liver of FSP1cre-*Pparb/d^−/−^* mice, which results in different protein levels and/or activities between the two genotypes, as reported above.

## 3. Discussion

We demonstrated through double immunofluorescence staining that CD11b^+^CD68^−^ liver macrophages, but not in other non-parenchymal hepatic cells, express FSP1. We further showed, using flow cytometry, that 14.5% of the bone marrow-derived cells express FSP1, of which 57% of these cells are CD11b^+^ cells. Hence, our results support an earlier observation of FSP1-positive cells in a subpopulation of inflammatory Kupffer cells/macrophages in injured livers [31].

The liver of FSP1cre-*Pparb/d^−/−^* mice is characterized by a change in hepatic lipid metabolism in 2-day-old pups and young mice. Histological analyses revealed that the microvesicular phenotype in the liver of FSP1cre-*Pparb/d^−/−^* was due to more and larger intracellular lipid droplets. This phenotype appeared in the livers of P2 and week 4 FSP1cre-*Pparb/d^−/−^* mice compared to control livers. Hence, we propose that the alteration of lipid metabolism in the liver of FSP1cre-*Pparb/d^−/−^* mice occurs in the early days of life, especially before and around weaning. As these FSP1cre-*Pparb/d^−/−^* pups aged, the lipid accumulation phenotype disappeared. Interestingly, one of our previous studies revealed that lipid accumulation in the liver of P2 *Ppara* knock out mice [36] and hepatocyte-specific deletion of *Ppara* resulted in impairment of fatty acid catabolism which caused hepatic lipid accumulation [37]. In the present study, this lipid accumulation phenotype was seen in the P2 and week 4 FSP1cre-*Pparb/d^−/−^* livers, although the underlying cause was different than *Ppara* deletion. This phenotype gradually disappeared in week 8.

Alteration in lipid levels can be due to changes in lipid biosynthesis or catabolism. Between P2 and week 4, there was a 70% reduction in triglyceride levels in both genotypes. However, the level of triglycerides at week 8 of age was similar to that of week 4. Therefore, we speculated that the significant difference in total lipids observed in the liver of FSP1cre-*Pparb/d^−/−^* mice at P2 and week 4 was probably associated with triglyceride uptake from milk fat. This would explain the significantly higher triglyceride level observed at P2 and week 4 of age and the similar triglyceride level in the livers of both genotypes at week 8 when the young mice were now fed on normal chow diet after weaning at P21. SREBP1c and SREBP2 are activators of the complete program of fatty acid and cholesterol synthesis in the liver [38]. We did not find any alteration in the cholesterol synthesis pathway in FSP1cre-*Pparb/d^−/−^* livers, which would impact total lipid levels at P2 and week 4. We noticed that the total cholesterol and free cholesterol levels were highest at P2, while cholesterol ester levels were highest at week 4 in both analyzed genotypes. Our qPCR results confirmed that there was no alteration in the expression of the cholesterol synthesis genes, which differed from the observation from the P2 *Ppara* knockout liver, which presented higher cholesterol ester levels [36]. The glucocorticoid receptor-PPARα axis that prepares neonates for milk lipid catabolism was impaired in the *Ppara* knockout liver. The deletion of *Ppara* drastically reduces fatty acid oxidation, leading to fat accumulation. Our results suggest that the mechanism of hepatic lipid accumulation in the FSP1cre-*Pparb/d^−/−^* liver is different, as already mentioned above.

The higher total lipids level in P2 and week 4 FSP1cre-*Pparb/d^−/−^* livers was attributed to a higher level of triglycerides, most likely due to an enhanced triglyceride synthesis. This alteration in triglyceride synthesis disappeared by week 8 after birth, which was in concordance with no difference in the levels of total lipids and triglycerides, as well as the number and size of lipid droplets. Remarkably, our qPCR results revealed that *Pparb/d* expression was lower in P2 FSP1cre-*Pparb/d^−/−^* liver. A lower *Pparb/d* expression in the liver is usually detected under the condition of higher *Ppara* expression and β-oxidation activity [39]. There was indeed an upregulation of genes involved in fatty acid β-oxidation, but also in fatty acid and triglyceride synthesis in FSP1cre-*Pparb/d^−/−^* livers. These results were supported by the protein expression analysis that confirmed the upregulation of these processes in P2 FSP1cre-*Pparb/d^−/−^* liver. However, the changes in gene expression were not seen anymore after weaning. We propose that the fat-rich milk diet of the pups promotes the lipid metabolism phenotype observed in FSP1cre-*Pparb/d^−/−^* liver. This phenotype would progressively disappear after the transition to a carbohydrate-rich diet.

**Figure 8:**
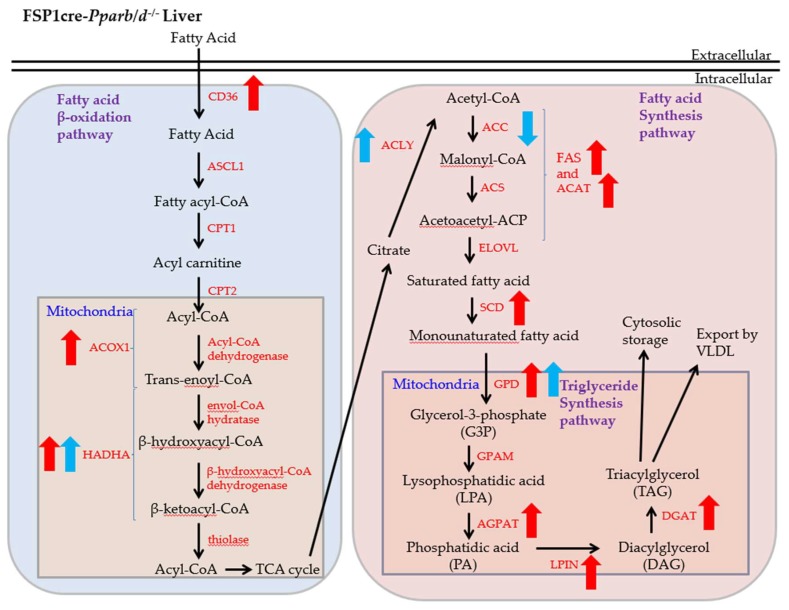
Overview of lipid metabolism, showing fatty acid β-oxidation, fatty acid synthesis, and triglyceride synthesis pathways affected in FSP1cre-*Pparb/d^−/−^* liver. Enzymes in the pathways are labeled in red, and RNA expression of those tested in this study and upregulated are indicated by a red arrow. Protein expression of HADHA and GPD, and ACLY and ACC activities, tested in this study are indicated by a blue arrow. For ACC, the down orientation of the blue arrow indicates a reduction of phospho-ACC in the liver of FSP1cre-*Pparb/d^−/−^* mice, which should result in a more active enzyme. Red arrow: RNA expression of the enzymes tested; blue arrow: protein expression of the enzyme tested.

The upregulation of the fatty acid β-oxidation pathway was somewhat unexpected in a situation of lipid accumulation. Fatty acid β-oxidation results in the production of acyl-CoA and then citrate via the TCA cycle (Figure 8). In the FSP1cre-*Pparb/d^−/−^* liver, there was an increase in phospho-ACLY, which would enhance citrate hydrolysis to increase the level of acetyl-CoA. In parallel, phospho-ACC was decreased in the P2 FSP1cre-*Pparb/d^−/−^* liver, enhancing the carboxylation of acetyl-CoA to malonyl-CoA to fuel fatty acid and triglyceride synthesis. Therefore, we hypothesized that the upregulation of fatty acid β-oxidation would participate in a higher level of triglycerides at P2 in the FSP1cre-*Pparb/d^−/−^* liver. The TCA cycle activity in the P2 FSP1cre-*Pparb/d^−/−^* liver remains to be determined. Taken together, we propose that FSP1cre-*Pparb/d^−/−^* mice, whose liver accumulates fat during the early days of life, maybe a useful animal model to study juvenile NAFLD. NAFLD is currently the most prevalent form of chronic liver disease, affecting up to 20% of the general pediatric population [40].

A limitation of this study is the lack of unveiling of the mechanism by which *Pparb/d* deletion in FSP1-expressing non-parenchymal hepatic cells leads to fat accumulation in hepatocytes before weaning. Ongoing work is addressing this question.

## 4. Materials and Methods

### 4.1. Chemicals and Antibodies

Unless otherwise stated, all chemicals and antibodies were purchased from Sigma-Aldrich (St Louis, MO, USA), Abcam (Cambridge, UK), Cell Signaling (Danvers, MA, USA), Kappa Biosystems (Wilmington, MA, USA), Roche Holding AG (Basel, Switzerland), Thermo Fisher Scientific (Waltham, MA, USA), Bio-Rad Laboratories (Hercules, CA, USA) and Cell Biolabs, Inc (San Diego, CA, USA), hematoxylin, eosin, ethanol, xylene, isopropanol, chloroform, NaCl, and HCl were obtained from Merck KGaA (Darmstadt, Germany).

### 4.2. Animals

Colonies of *Pparb/d^fl/fl^* and FSP1cre-*Pparb/d^−/−^* mice on a (C57BL/6J×Sv129)×BALB/c-Tg mixed background were provided by Associate Professor Tan Nguan Soon’s laboratory (NTU, Singapore) and bred in our animal facility. They were generated in Tan’s laboratory using *Pparb/d^fl/fl^* mice provided by Prof. Walter Wahli [41]. Mice were maintained with close monitoring in a specific pathogen-free facility at 25 °C on a 12-h/12-h light-dark cycle. They were kept in microisolator cages and fed on standard rodent chow diet and given water ad libitum. All experimental protocols were approved by the Nanyang Technological University-Institutional Animal Care and Use Committee (ARF-CSB-E0004, 10 October 2013 and ARF-CSB-E0007, 25 August2016) in Singapore and followed the NIH Guide for the Care and Use of Laboratory Animals. To avoid estrous cycle variations that might cause confounding actors in data interpretation, only male mice were used in all experiments and these mice were litter mates.

### 4.3. Genotyping Analysis

*Pparb/d^fl/fl^* and FSP1cre-*Pparb/d^−/−^* mice used at week 4 and 8 of age were weaned and genotyped at three weeks of age as previously described [42]. Genotyping via tail nicking of P2 of age mice was carried out together with the harvesting of livers. Prior to ear biopsy or tail cut, mice were anesthetized via intraperitoneal injection of ketamine: xylazine (100 mg: 10 mg per kg body weight) dissolved in 0.9% saline solution. An ear biopsy of approximately 2 × 2 mm^2^ or 2 mm of the tail cut was taken. Extraction of DNA from the biopsy was carried out with the KAPA HotStart Mouse Genotyping Kit (Kappa Biosystems, Wilmington, MA, USA), based on the suggested protocol from the kit.

Mice were genotyped by a multiplex polymerase chain reaction (PCR) assay, whereby the *Pparb/d^fl/fl^* allele was detected using primer PBX10 (5′-GCAGCTGCTCAGCTGCCTGC-3′) and primer AB008 (5′-ATGCCGAGTGCCAGGCACTTCTGGAAG-3′). The mutant allele was detected using primer PBX10 (see above) and primer AB021 (5′-GGACCCCGTAGTGGAAGCCCGAGGCC-3′). The FSP1 control allele was detected using primer 1 (5′-CTAGGCCACAGAATTGAAAGATC-3′) and primer 2 (5′-GTAGGTGGAAATTCTAGCATCAT-3′). The FSP1-cre allele was detected using primer 3 (5′-GCGGTCTGGCAGTAAAAACTATC-3′) and primer 4 (5′-GTGAAACAGCATTGCTGTCACTT-3′). The primers were synthesized by Integrated DNA Technologies, Singapore. The genomic DNA was amplified by PCR using KAPA HotStart Mouse Genotyping Kit (Kappa Biosystems, Wilmington, MA, USA). The protocol is provided in Appendix A. PCR products were subsequently analyzed by electrophoresis on 1.5% agarose gels.

Male P2 mice were determined by the sex-determining region of the Y chromosome. SRY allele was detected using primer SRY forward primer (5′-TTGTCTAGAGAGCATGGAGGGCCATGTCAA-3′) and SRY reverse primer (5′-CCACTCCTCTGTGACACTTTAGCCCTCCGA-3′). The primers were synthesized by Integrated DNA Technologies, Singapore. The genomic DNA was amplified by PCR using KAPA HotStart Mouse Genotyping Kit (Kappa Biosystems, Wilmington, MA, USA). The protocol is provided in Appendix A. PCR products were subsequently analyzed by electrophoresis on 1.5% agarose gels.

### 4.4. Histology

Specimens taken from the liver left lateral lobe were briefly rinsed in 1× phosphate buffer saline (PBS; pH 7.4), which comprises 2.7 mM KCL, 137 mM NaCl, 2 mM KH_2_PO_4_, and 10 mM Na_2_HPO_4_, before being fixed overnight with 4% paraformaldehyde/PBS (pH 7.4) at 4 °C. The tissue was subjected to dehydration procedure in 50% EtOH, 60% EtOH, 70% EtOH, 80% EtOH, 90% EtOH, twice in 100% EtOH, EtOH/xylene, twice in xylene at 1 h each. The dehydrated liver sections were infiltrated with molten paraffin at 60 °C overnight before embedding fixing into paraffin blocks. The *Pparb/d^fl/fl^* and FSP1cre-*Pparb/d^−/−^* livers were sectioned at 5 µm thickness. For hematoxylin and eosin (H&E) staining, the sections were deparaffinized, rehydrated, stained with hematoxylin for 3 min, and differentiated in 0.1% HCl for 2 s prior counterstaining with eosin for 27 s. For Oil red O staining preparation, the fresh liver samples from the left lateral lobe were collected and embedded in OCT freezing medium (Leica Microsystems, Wetzlar, Germany). A stock solution of Oil red O was prepared by dissolving 0.5 g of Oil red O powder (Sigma-Alrich, St Louis, MO, USA) in 500 mL isopropanol. 50 mL of 60% Oil red O stock solution was diluted with 20 mL of deionized water to prepare the working solution. The frozen specimens were cryosectioned at 8 µm thickness and stained with Oil Red O working solution for 10 min and followed by counterstaining with hematoxylin. Images were acquired using the ZEN 2012 LE software and Axio Scan.Z1 slide scanner microscope with 10× and 20× objective lenses (Carl Zeiss AG, Oberkochen, Germany).

### 4.5. Double Immunofluorescence Staining

For double immunofluorescence staining, antibodies against CD68 (#137002) (San Diego, California, USA), GFAP (#ab68428), CD11b (#ab75476), S100A4 (#ab27957) (Abcam, Cambridge, MA, USA) and CD146 PE (#130102319) (Miltenyi Biotec, Bergisch Gladbach, Germany) were used. All other fluorophore-conjugated secondary antibodies were acquired from Life Technologies (Carlsbad, CA, USA). For flow cytometry analyses, antibodies against CD45 FITC (#553080), CD11b PerCP-Cy5.5 (#550993), CD16/32 (#553142) (BD Biosciences, Franklin Lakes, NJ, USA), CD146 PE (#130102319), CD68 APC (#130102585) (Miltenyi Biotec, Bergisch Gladbach, Germany), GFAP (#ab68428) and cytokeratin18 (FITC) (#ab52459) (Abcam, Cambridge, MA, USA) were used. All other fluorophore-conjugated secondary antibodies were acquired from Life Technologies.

Prior to double immunofluorescence staining, the sections were melted at 60 °C before the rehydrating procedure in 2 times in xylene, EtOH/xylene, 2 times in 100% EtOH, 90% EtOH, 80% EtOH, 70% EtOH, 60% EtOH, and 50% EtOH at 5 min each. The tissue sections were subjected to antigen retrieval procedure in 10 mmol/L sodium citrate buffer (pH 6.0) at 95 °C for 20 min. The slides were washed thrice with PBS before blocking with 5% BSA for 1 h. Tissue sections were incubated with either anti-GFAP (1:100), or anti-CD11b (1:100), or anti-CD68 (1:100), or anti-CD146 (1:100) in 0.5% BSA overnight at 4 °C. Except for the tissue sections incubated with anti-CD146 or anti-CD68, the rest of the tissue sections were incubated with secondary goat anti-rabbit Alexa Fluor 488 antibodies (1:200) in 0.5% BSA with incubation for 1 h at room temperature in the dark. Tissue sections incubated with CD68 were incubated with secondary goat anti-rat Alexa Fluor 488 antibodies (1:200) in 0.5% BSA with incubation for 1 h at room temperature in the dark. Tissue sections were then incubated with anti-S100A4 (1:100) in 0.5% BSA overnight at 4 °C. Secondary goat anti-rabbit Alexa Fluor 594 antibodies (1:200) in 0.5% BSA were added with incubation for 1 h at room temperature in the dark. The tissue sections were counterstained with DAPI (Vectashield, Burlingame, CA, USA). Between each step, slides were washed thrice with PBS. Immunofluorescent images were taken using a Carl Zeiss (Thornwood, NY, USA) confocal microscope LSM 710meta using a Plan-Apochromat × 63/1.4 oil DIC objective, and ZEN 2012 LE software with constant exposure and gain.

### 4.6. Isolation of Hepatic Cells

P2 *Pparb/d^fl/fl^* and FSP1cre-*Pparb/d^−/−^* livers were harvested and briefly rinsed with 1× PBS (pH 7.4) prior they were minced into smaller pieces. Each liver was digested in 2 mL HBSS solution with calcium and magnesium (#14025092) (Life Technologies) with 200 U/mL collagenase type IV (#17104019) (Life Technologies) for 30 min in 37 °C thermo-shaker at 120 rpm. The remaining liver tissue pieces were disaggregated with the use of the 18-gauge blunt needle syringe. Upon confirmation of the genotype and gender, the cell suspension was pooled and filtered through the 100 µm cell strainer into a 15 mL Eppendorf tube and topped up to 15 mL with Dulbecco’s modified Eagle’s medium (DMEM) containing 10% fetal bovine serum (#16000036; Life Technologies). The cell suspension was centrifuged at 300 *g* for 5 min. The supernatant was discarded, and the cell pellet resuspended in HBSS solution with calcium and magnesium. The cell suspension was centrifuged at 300 *g* for 5 min; the supernatant was discarded prior adding 5 mL of red blood cell lysis buffer (1.5 M NH_4_Cl, 0.1 M NaHCO_3_, and 0.01 M EDTA) for 10 min on ice before filtering through the 100-µm cell strainer. The cell suspension was washed 2 times before proceeding to flow cytometric analysis.

### 4.7. Flow Cytometry

Cells from *Pparb/d^fl/fl^* and FSP1cre-*Pparb/d^−/−^* livers were then subjected to LIVE/DEAD™ Fixable Blue Dead Cell Stain Kit (#L23105) (Life Technologies) prior subjecting to ice cold 0.02% saponin + 2% FBS in PBS solution for 10 min. Thereafter, the cells were blocked with anti-CD16/32 (1:50) (#553142, BD, Franklin Lakes, NJ, USA) on ice for 10 min. Thereafter, for isolation of CD45+FSP1+CD11b+ cells, the cells were treated with anti-CD45 PE-Texas Red (#ab51482, Abcam, Cambridge, MA, USA), CD11b PerCP-Cy5.5 (#550993, BD, Franklin Lakes, NJ, USA), S100A4 (#ab27957, Abcam, Cambridge, MA, USA) antibodies respectively at a concentration (1:100) and incubated on ice in the dark for 15 min. The cells were washed prior adding secondary antibody conjugated with Alexa Fluor 594 (#A-21207, Life Technologies) for S100A4+ cells at a concentration (1:200) in 2% FBS in PBS solution on ice in the dark for 15 min. For isolation of hepatocytes, the cells were treated with Cytokeratin18 FIT-C (#ab52459, Abcam, Cambridge, MA, USA) at a concentration (1:100) and incubated on ice in the dark for 15 min. Stained cells were washed and kept on ice in PBS solution with 2% FBS prior analysis and isolation via a Becton Dickinson (BD, Franklin Lakes, NJ, USA) BD FACSAria™ Cell Sorter. Analyses of flow cytometry results were performed with the BD FACSDiva™ software (version 8.0, BD, Franklin Lakes, NJ, USA).

### 4.8. Quantification of Total Lipids, Total Cholesterol, and Triglycerides

Ten sets of tissue samples were used for each genotype and each age group. Total lipids, total cholesterol and triglyceride levels in *Pparb/d^fl/fl^* and FSP1cre-*Pparb/d^−/−^* livers were determined with quantitative enzymatic assays using the Lipid Quantification Kit STA-613 (Cell Biolabs, Inc, San Diego, CA, USA), Total Cholesterol Assay Kit STA-384 (Cell Biolabs, Inc, San Diego, CA, USA) and Serum Triglyceride Quantification Kit STA-396 (Cell Biolabs, Inc, San Diego, CA, USA), respectively. Prior measurement of total lipids, the lipids in the livers were extracted using the Lipid Extraction Kit STA-612 (Cell Biolabs, Inc, San Diego, CA, USA). The procedures were in accordance with the protocols provided by the manufacturer. The enzymatic reactions of the Lipid Quantification Kit and Total Cholesterol Assay Kit generated a dye that absorbs light at 540 nm, whereas enzymatic reactions from the Serum Triglyceride Quantification Kit generated a dye that absorbs light at 570 nm. A standard curve was generated for each individual assay, using the serial dilutions of the standard solution provided in each kit. Using the optical density values at the specific absorbance wavelength against the respective concentrations of standards, the standard curves were generated using Microsoft^®^ Office Excel 2010 and best fitted to a linear function with an R2 ≥ 0.98. The calculated total lipids level was normalized with the mass of liver tissue, while the calculated triglyceride, total cholesterol, cholesterol ester, and free cholesterol levels were normalized with the total protein determined with the Bradford assay protein assay #5000006 (Bio-Rad Laboratories, Hercules, CA, USA).

### 4.9. Total RNA Extraction and Reverse Transcription

50–100 mg of liver tissue in 1 mL of TrIzol^®^ Reagent (Invitrogen, Carlsbad, CA, USA) was processed by TissueLyser II (Qiagen, Hilden, Germany). Each homogenate was subjected to centrifugation at 12,000 *g* for 10 min at 4 °C to remove the insoluble materials. The soluble RNA in the supernatant was transferred to a new tube. 0.2 mL of chloroform was added and mixed vigorously for 15 s, prior incubation at room temperature for 5 min. The samples were centrifuged at 12,000 *g* for 15 min at 4 °C. Approximately 400 µL of the colorless RNA-containing aqueous phase was transferred to a fresh tube and an equal volume of 70% ethanol was added and vortexed. These exacted RNA samples were further purified using the PureLink RNA Mini Kit (Invitrogen, Carlsbad, CA, USA) accompanied with a DNase I treatment (Roche Applied Science, Penzberg, Upper Bavaria, Germany), according to the protocol from the manufacturer. The extracted RNA was spectrophotometrically measured, and quality was determined by absorbance ratios at 260 nm/280 nm and 260 nm/ 230 nm through Nanodrop Spectrophotometer (Thermo Fisher Scientific, Waltham, MA, USA). Every one microgram of RNA was reverse transcribed with the iScript™ cDNA Synthesis Kit (Hercules, CA, USA), according to the manufacturer’s protocol. The cDNA products were diluted 5 times and stored at −20 °C.

### 4.10. Quantitative Real-Time PCR

Ten sets of tissue samples were used for each genotype and each age group. The sequence of the primer pairs was obtained from the NCBI Primer Blast available online at https://www.ncbi.nlm.nih.gov/tools/primer-blast/ and summarized in Appendix B. Quantitative q(PCR) was performed on a StepOnePlus Real-Time PCR System (Thermo Fisher Scientific, Waltham, MA, USA) using KAPA FAST qPCR kit (Kappa Biosystems, Wilmington, MA, USA). Gadph (forward primer sequence: 5′-AGGTCGGTGTGAACGGATTTG-3′; reverse primer sequence: 5′-TGTAGACCATGTAGTTGAGGTCA-3′) was used as the normalizing housekeeping gene.

### 4.11. Western Blot and Densitometric Analysis

Seven sets of tissue samples were used for each genotype and each age group. Protein was extracted from each left lateral liver lobe, 0.1 g of tissue in 300 µL of RIPA Lysis and Extraction Buffer (#89900, Thermo Fisher Scientific, Waltham, MA, USA) with cOmplete™, Mini, EDTA-free Protease Inhibitor Cocktail (#11836170001, Roche Applied Science, Penzberg, Upper Bavaria, Germany) and PhosSTOP™ (#Phoss-Ro, Roche Applied Science, Penzberg, Upper Bavaria, Germany), using TissueLyser II (Qiagen, Hilden, Germany). The cell lysate was centrifuged at 12,000 *g* at 4 °C for 10 min to sediment the cell debris and segregate the lipids. The infranatant was carefully aspirated into a fresh tube. Equal amounts of protein extracts (40 µg) were resolved by 12% SDS-polyacrylamide gel electrophoresis and electrotransferred onto a polyvinylidene fluoride membrane. Membranes were processed as described by the manufacturer of antibodies, and chemiluminescence was detected by SuperSignal™ West Pico PLUS Chemiluminescent Substrate (#34580, Thermo Fisher Scientific, Waltham, MA, USA) at room temperature for 5 min. Beta-tubulin (#ab6046, Abcam, Cambridge, MA, USA), beta-actin (#sc-47778 HRP) or U2AF65 (#sc-48804, Santa Cruz, Dallas, TX, USA) was used to check for equal loading and transfer. PPARβ/δ (#sc-7197, Santa Cruz, Dallas, TX, USA), *ACC* (#3676), phospho-*ACC* (#11818), *ACLY* (#4332), phospho-*ACLY* (#4331) (Cell Signaling Technology, Danvers, MA, USA), HADHA (#ab203114) and GPD2 (#ab188585) (Abcam, Cambridge, MA, USA) were used. Densitometric analysis was carried out using ImageJ software (version 1.46r; NIH, Bethesda, MD, USA). Band intensity values indicate the average of 7 samples of each genotype. Normalization of protein levels was carried out to the loading control of Beta-tubulin or beta-actin (e.g., *ACLY*/β-tubulin). Phosphorylated protein levels were further normalized to their respective non-phosphorylated counterparts (e.g., p-*ACLY*/total *ACLY*).

### 4.12. Statistical Analysis

Values are shown as mean±standard error of the mean (SEM). The two-tailed Mann-Whitney test and two-way ANOVA were performed using GraphPad Prism (version 4.00) software. Two-tailed Mann-Whitney test is a non-parametric test that was used to compare two sample means that come from the same population, and used to test whether the two sample means are equal or not, without assuming a gaussian distribution. For quantification of total lipids, total cholesterol, and total triglyceride levels, two-way ANOVA was also used to compare the mean differences between the ages (P2, 4 weeks, 8 weeks) of *Pparb/d^fl/fl^* and FSP1cre-*Pparb/d^−/−^* livers. In both statistical tests, *P* < 0.05 was considered statistically significant.

## Supplementary Materials

Supplementary materials can be found at https://www.mdpi.com/1422-0067/20/20/5115/s1.

## Abbreviations

ACAA2	acetyl-CoA acyltransferase 2
ACAT1	acetyl-CoA acetyltransferase 1
ACC	acetyl-CoA synthetase
ACLY	acyl-CoA oxidase 1
ACS	acetyl-CoA synthetase
ACOX1	acyl-CoA oxidase 1
AGPAT3	1-acylglycerol-3-phosphate O-acyltransferase 3
AMPK	AMP-activated protein kinase
CYP51	cytochrome P450 family 51 subfamily A member 1
DGAT1	diacylglycerol O-acyltransferase 1
DHCR7	7-dehydrocholesterol reductase
FAS	fatty acid synthase
FSP1	fibroblast-specific protein 1
GFAP	glial fibrillary acidic protein
GPD1	glycerol 3-phosphate dehydrogenase 1
GPD2	glycerol 3-phosphate dehydrogenase 2
H&E	hematoxylin and eosin
HADHA	hydroxyacyl-CoA dehydrogenase
HMGCS1	hydroxymethylglutaryl-CoA synthase
HSC	hepatic stellate cell
IL4	interleukin 4
IL13	interleukin 13
IgG	immunoglobulin G
LPIN3	lipin 3
LSEC	liver sinusoidal endothelial cell
NAFLD	non-alcoholic fatty liver disease
NASH	non-alcoholic steatohepatitis
PLIN5	perilipin 5
PPAR	peroxisome proliferator-activated receptor
ROS	reactive oxygen species
SCD1	stearoyl-CoA desaturase 1
SREBP1c	sterol regulatory element-binding protein 1
SREBP2	sterol regulatory element-binding protein 2
TG	triglycerides
TZDs	thiazolidinediones

## Appendix A

***Pparb/d^fl/fl^* Genotyping Protocol**	
**PCR Reaction mixture**	**PCR program setting**
1.2 µL PBX10	95 °C 3 min
0.6 µL AB008	
0.6 µL AB021	30 cycles:
8.1 µL Water	94 °C 15 s
12.5 µL 2× KAPPA2G Fast (HotStart) genotyping mix with dye	68 °C 10 s
	72 °C 20 s
	
	72 °C 10 min
	4 °C hold
**FSP1cre-*Pparb/d^−/−^* Genotyping Protocol**	
**PCR Reaction mixture**	**PCR program setting**
0.375 µL *Fsp1* Ctrl Forward	94 °C 3 min
0.375 µL *Fsp1* Ctrl Reverse	
1.25 µL *Fsp1* Forward	30 cycles:
1.25 µL *Fsp1* Reverse	95 °C 15 s
8.25 µL Water	59.2 °C 30 s
12.5 µL 2× KAPPA2G Fast (HotStart) genotyping mix with dye	72 °C 10 s
1.0 µL template	
	72 °C 10 min
	4 °C hold
***Sry* Genotyping Protocol**	
**PCR Reaction mixture**	**PCR program setting**
1 µL *Sry* Forward	94 °C 5 min
1 µL *Sry* Reverse	
3.5 µL Water	33 cycles:
7.5 µL 2× KAPPA2G Fast (HotStart) genotyping mix with dye	94 °C 30 s
1.0 µL template	60 °C 30 s
	72 °C 45 s
	
	72 °C 10 min
	4 °C hold

## Appendix B

**Gene**	**Primer Sequence**
*Acaa2*	Forward (5′-3′) TCTTGACCCCAGCAAAACCA
	Reverse (5′-3′) CCACCTCGACGCCTTAACTC
*Acc*	Forward (5′-3′) GACAACACCTGTGTGGTGGA
	Reverse (5′-3′) GAGGTTGGAGGCAAAGGACA
*Acat1*	Forward (5′-3′) AATGCTGGAGATTGACCCCC
	Reverse (5′-3′) CGGGCTCCAGACATCCCAAT
*Acly*	Forward (5′-3′) GTCCCAAGTCCAAGATCCCTG
	Reverse (5′-3′) TGTGATCCCCAGTGAAAGGG
*Acox1*	Forward (5′-3′) GAGCAGCAGGAGCGTTTCTT
	Reverse (5′-3′) CAGGACTATCGCATGATTGGAAG
*Agpat3*	Forward (5′-3′) TACTGCGAAGGAACACGCTT
	Reverse (5′-3′) TCTTGCAGGGCATCCTTCTC
*Cd36*	Forward (5′-3′) TGATACTATGCCCGCCTCTCC
	Reverse (5′-3′) TTTCCCACACTCCTTTCTCCTCTA
*Cyp51*	Forward (5′-3′) CGTCTACCTGTTCCGTCTCG
	Reverse (5′-3′) ATGTGGTGGACTTTTCGCTC
*Dgat1*	Forward (5′-3′) TAGAAGAGGACGAGGTGCGAG
	Reverse (5′-3′) GTAGAGACAGCTTTGGCCTTGA
*Dhcr7*	Forward (5′-3′) ATGGCTTCGAAATCCCAGCA
	Reverse (5′-3′) GAACCAGTCCACTTCCCAGG
*Fas*	Forward (5′-3′) AGTGTCCACCAACAAGCG
	Reverse (5′-3′) GATGCCGTCAGGTTTCAG
*Fsp1*	Forward (5′-3′) CTCTCTTGGTCTGGTCTCAAC
	Reverse (5′-3′) AACTTGTCACCCTCTTTGCCT
*Gpd1*	Forward (5′-3′) CATCACGACCTGCTATGGGG
	Reverse (5′-3′) GCTGCTCAATGGACTTTCCAG
*Gpd2*	Forward (5′-3′) ACTCCGTTCTGGCTGGAGGAT
	Reverse (5′-3′) CATATGCCAGGCTCACTTGCTTC
*Hadha*	Forward (5′-3′) TGCTCAAGATGGTGGCGTCC
	Reverse (5′-3′) AATGCAGCCTCTGGAGCGTAG
*Hmgcs1*	Forward (5′-3′) TCCCCTTTGGCTCTTTCACC
	Reverse (5′-3′) GGGCAACGATTCCCACATCT
*Lpin3*	Forward (5′-3′) ACAGCTCATTGTACGTGGCA
	Reverse (5′-3′) ATCTCCACATGGCTTTCCCG
*Plin5*	Forward (5′-3′) GCCATCAGACATGGTGGTGA
	Reverse (5′-3′) CCTCAGTCATGGGCAGGAAG
*Ppara*	Forward (5′-3′) TACTGCCGTTTTCACAAGTGC
	Reverse (5′-3′) AGGTCGTGTTCACAGGTAAGA
*Pparb/d*	Forward (5′-3′) CGGCAGCCTCAACATGG
	Reverse (5′-3′) AGATCCGATCGCACTTCTCATAC
*Pparg1*	Forward (5′-3′) TGTGAGACCAACAGCCTGAC
	Reverse (5′-3′) CCGCTTCTTTCAAATCTTGTCTGT
*Scd1*	Forward (5′-3′) GCCCACATGCTCCAAGAGAT
	Reverse (5′-3′) GGGCACTGTCTTCACCTTCT
*Srebp1c*	Forward (5′-3′) AAGCGCTACCGGTCTTCTATC
	Reverse (5′-3′) TGTGCACTTCGTAGGGTCAG
*Srebp2*	Forward (5′-3′) TGGGCGATGGATGAGAGCAG
	Reverse (5′-3′) AACTGTAGCATCTCGTCGATGTC
