# Supplementary material for: The Potential of the FSP1cre-Pparb/d−/− Mouse Model for Studying Juvenile NAFLD
Source: Int J Mol Sci. 2019 Oct 15;20(20):5115. doi: 10.3390/ijms20205115 (PMC6830345; doi:10.3390/ijms20205115)
Supplement: Supplementary file 1 [file ijms-20-05115-s001.pdf]

**Supplementary Materials**

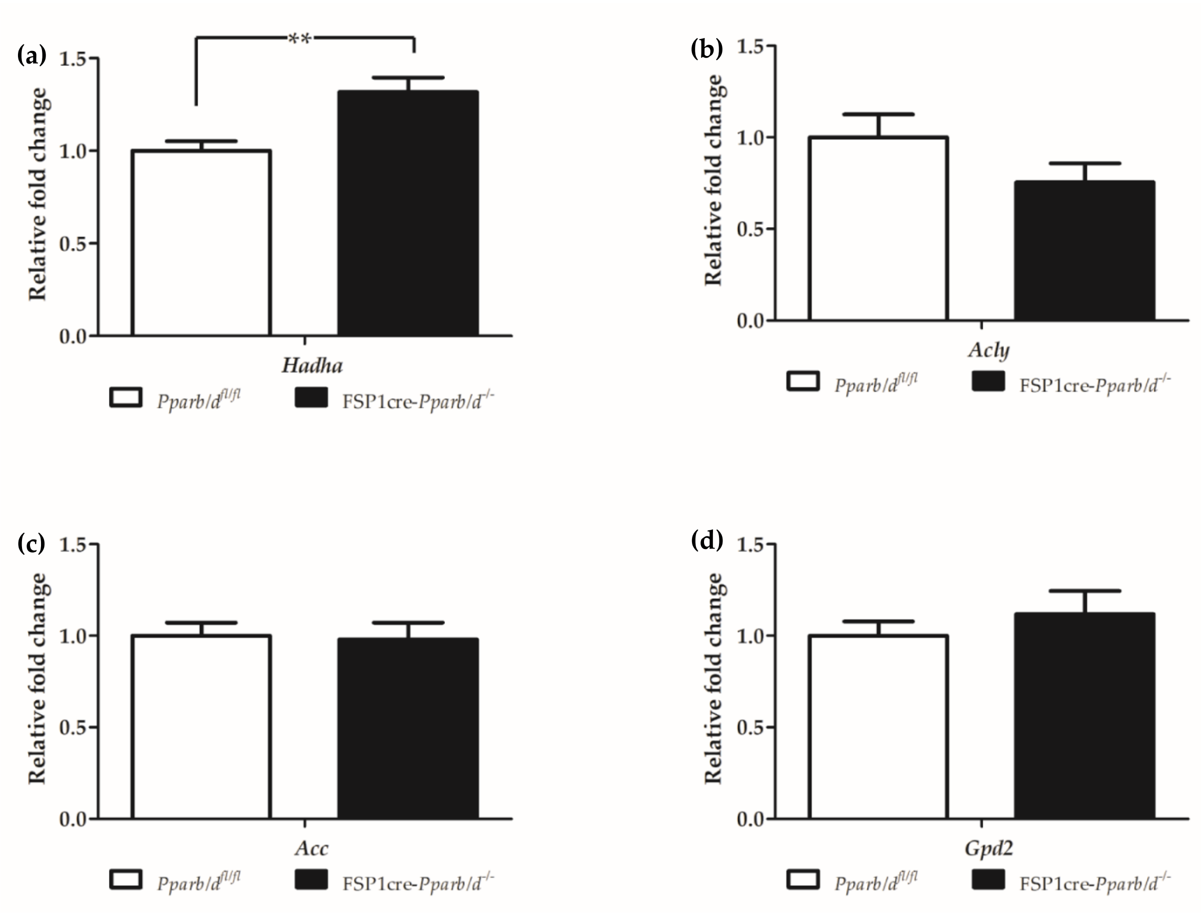


**Figure SM1.** Effect of *Pparb/d* deletion on liver gene expression at P2. Relative fold change in the mRNA levels of specific genes in livers of *Pparb/d*$^{fl/fl}$ and FSP1cre-*Pparb/d*$^{-/-}$ mice as determined by real-time qPCR. **(a)** Expression of *Hadha* (presented differently in **Figure 6b**). **(b)** *Acly*. **(c)** *Acc*. **(d)** *Gpd2*. Values are normalised to the expression of *Gapdh*. Normalised values from controls were arbitrarily assigned a value of 1. Two-tailed Mann-Whitney test with values shown as mean ± s.e.m, n = 9-10 biological replicates per group. ** $P < 0.01$; FSP1cre-*Pparb/d*$^{-/-}$ vs *Pparb/d*$^{fl/fl}$ controls.

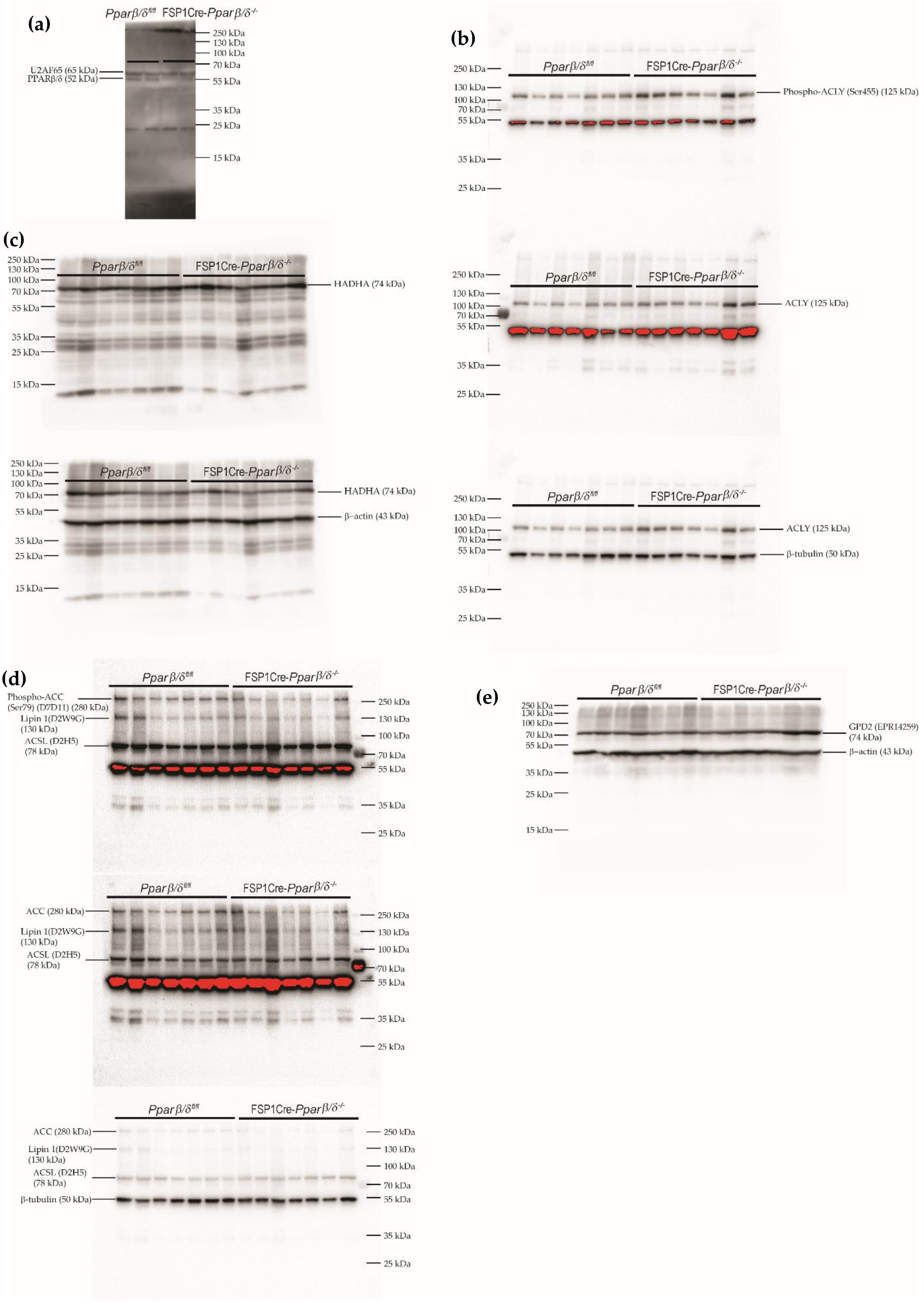


**Figure SM2.** Images of the original Western Blots. **(a)** Immunoblot analysis of PPARβ/δ in P2 *Pparb/d*$^{fl/fl}$ and FSP1cre-*Pparb/d*$^{-/-}$ whole livers (2 livers for each genotype) with U2AF65 as loading and transfer control. **(b)** Immunoblot analysis of phospho-ACLY and ACLY in P2 *Pparb/d*$^{fl/fl}$ and FSP1cre-*Pparb/d*$^{-/-}$

whole livers (7 livers for each genotype) with β-tubulin as loading and transfer control. **(c)** Immunoblot analysis of HADHA in P2 *Pparb/d*$^{fl/fl}$ and FSP1cre-*Pparb/d*$^{-/-}$ whole livers (7 livers for each genotype) with β-actin as loading and transfer control. **(d)** Immunoblot analysis of phospho-ACC and ACC in P2 *Pparb/d*$^{fl/fl}$ and FSP1cre-*Pparb/d*$^{-/-}$ whole livers (7 livers for each genotype) with β-tubulin as loading and transfer control. **(d)** Immunoblot analysis of phospho-ACLY and ACLY in P2 *Pparb/d*$^{fl/fl}$ and FSP1cre-*Pparb/d*$^{-/-}$ whole livers (7 livers for each genotype) with β-tubulin as loading and transfer control. **(e)** Immunoblot analysis of GPD2 in P2 *Pparb/d*$^{fl/fl}$ and FSP1cre-*Pparb/d*$^{-/-}$ whole livers (7 livers for each genotype) with β-actin as loading and transfer control. The red bands indicate oversaturation of signals, which are not used in quantification of respective protein expression levels.
